# PocketPipe: A computational pipeline for integrated Pocketome prediction and comparison

**DOI:** 10.6026/97320630015295

**Published:** 2019-04-15

**Authors:** Samdani Ansar, Anupriya Sadhasivam, Umashankar Vetrivel

**Affiliations:** 1 1Centre for Bioinformatics, Kamalnayan Bajaj Institute for Research in Vision and Ophthalmology, Vision Research Foundation, SankaraNethralaya, Chennai - 600 006, Tamil Nadu, India; 2 2School of Chemical and Biotechnology, SASTRA Deemed University, Thanjavur, India

**Keywords:** Pocketome, pipeline, binding pocket

## Abstract

Functional characterisation of proteins often depends on specific interactions with other molecules. In the drug discovery scenario, the ability of a protein to bind with drug-like molecule with a high affinity is referred as druggability. Deciphering such druggable binding pockets on proteins plays an important role in structure-based drug designing studies. Moreover, availability of plethora of structural data poses a need automated pipelines which can efficiently integrate robust algorithms towards large-scale pocket identification and comparison. These pipelines have direct applicability on off-target analysis, drug repurposing and structural prioritization of drug targets in pathogenic microbes. However, currently there is a paucity of such efficient pipelines. Hence, by this study a highly optimized shell script based pipeline (PocketPipe) has been developed with seamless integration of robust algorithms namely, P2Rank (predicts binding sites based on machine learning) and PocketMatch-v2.1 (compares binding pockets by residue-based method), for pocketome generation and comparison, respectively. The process of input workflow and various steps carried out by PocketPipe and the output results are well documented in the operating manual. On execution, the pipeline features seamless operability, high scalability, dynamic file handling and results parsing. PocketPipe is distributed freely under GNU GPL license and can be downloaded at https://github.com/inpacdb/PocketPipe

## Background

In this post genomic era, a pocketome representing the entire druggable sites of an organism has become essential part of drug designing. Pocketome can be efficiently utilized for drug repurposing, target prediction, subtracting the overlapping drug binding pockets among host and infective organisms, elimination of off-target effects etc. 1. Hence, it becomes increasingly interesting to exploit this 'pocket space' as a kind of dictionary to accelerate modern in silico drug design processes. Earlier studies have discussed on the pocket space and drug design 2. 

Nevertheless, there is paucity in the availability of efficient software pipelines which integrates efficient tools for large-scale pocket prediction and comparison. Moreover, open source based automated pipelines serve as indispensable component of computer aided drug design 3, 4. Hence, by this study we have developed a shell script based pipeline named Pocketpipe, with seamless integration of efficient algorithms for large-scale pocketome generation and matching. Hence, P2Rank for pocket prediction and PocketMatch for pocket matching were selected for integration. P2Rank 5, predicts the ligand binding sites and its score is calculated as a sum of squared ligand ability scores of individual pocket points 6. PocketMatch-v.2.1 7 compares pair of sites based on the alignment of sorted sequence of distance between pairs of point representing sites. The choice of tools was based on ease automation, plausibility in pocket prediction, compatibility with shell scripting and adaptability with massive parallelization. The usage of this pipeline was also demonstrated in a study on Chlamydia trachomatis 8, however, the pipeline was not published. 

## Methodology

### Input:

**Figure 1:**
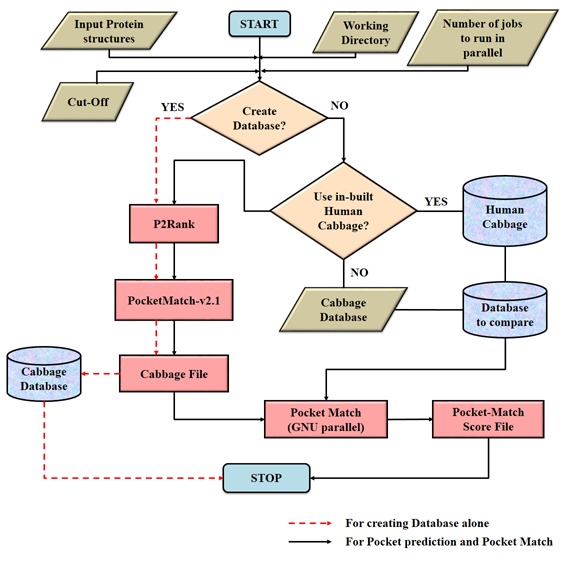
Flowchart representing the integrative automation of P2Rank and PocketMatch-v2.1 algorithms. The red dashed represents the processes involved in database creation, whilst, the solid black lines represents the processes for pocket prediction and pocket matching.

PocketPipe was developed as a shell script featuring dynamic file handling, parsing and GNU-parallel based parallelization and automated report generation. On invoking the pipeline in linux terminal, the user will be prompted to enter the path directory containing protein datasets (in .pdb format) and also a working directory. Subsequently, user needs to provide the number of CPUs to be used for pocket prediction. User also needs to choose the option whether the current run is meant to for creating a pocketome database, otherwise can skip directly to comparison for a pre-created pocketome. In case of create pocketome option, the pipeline triggers P2Rank and collates all the binding pockets in .pdb format, subsequently PocketMatch converts all .pdb files into a .cabbage file which is ready for pocketome comparison. If the user has a pre-created database as a single .cabbage file, one can skip this process and directly navigate to pocket comparison. Next the user will be prompted to provide the number of CPUs to be used for pocketome comparison. Here, the user also has to feed the PocketMatch cut-off values (=0.4, = 0.6, =0.8) to prioritize the nonoverlapping pockets among two datasets. On providing all these data, PocketPipe iteratively runs the processes and populates the results as a tab-delimited file (Figure 1). 

### Output:

For the input protein datasets, PocketPipe will predict the pockets using P2Rank. PocketPipe integrates the information for each protein from its respective .CSV files from P2Rank and parses the pocket residues from the corresponding structure(s) and reports as .pdb files. This feature is unique to PocketPipe, as manual parsing of huge datasets is highly tedious. For comparison of the predicted pockets, the .cabbage file of 50 predicted pockets/batch will be created into the pocket_match directory. Depending on the number of CPU to run in parallel, each batch of cabbage file will be run in parallel to perform pocket comparison with the specified database. Upon completion of the pocket comparison, the results will be appended in the working directory with the file name specified as Pocket_Match_raw.txt which contains pocket scores of all the comparison performed whilst, Below_cutoff.txt file captures the pocket scores within the user specified cut-off. 

## Caveats and future development:

PocketPipe is written in bash shell programming language, which can run in Linux OS with the installed dependencies P2Rank and PocketMatch-v.2.1. Currently, we are attempting to integrate all the available free open source pocket prediction and comparison tools for ease of use. 

## Conclusion

Computed aided drug design has become an indispensable part of Drug Discovery right from targeting microbes 9, 10 to disease conditions in humans. This poses a need for efficient open source based pipelines for efficient analysis. Hence, by this study, we contribute a computational pipelinewhich effectively performs pocketome creation and comparison through seamless integration of P2Rank and PocketMatch algorithms, respectively. Thus, PocketPipe shall form the essential part of computational toolkit of scientists working on pocketome research. 

## Conflict of Interest

Authors declare no conflict of interest.
